# “Attempting to protect self and patient”: A grounded theory study of error recovery by intensive care nurses

**DOI:** 10.1002/nop2.1719

**Published:** 2023-03-13

**Authors:** Tahereh Najafi Ghezeljeh, Mansoureh Ashghali Farahani, Fatemeh Kafami Ladani

**Affiliations:** ^1^ Nursing Care Research Center, School of Nursing and Midwifery Iran University of Medical Sciences Tehran Iran

**Keywords:** error recovery, grounded theory, intensive care unit, medical error, nurse

## Abstract

**Aim:**

The aim of this study was to explore the process of error recovery (ER) by nurses in intensive care unit (ICU).

**Design:**

This qualitative study was conducted in 2018–2020 using the grounded theory methodology.

**Methods:**

Participants were 20 staff nurses, head nurses and nursing managers recruited from the ICUs. Sampling was started purposively and continued theoretically. Data were collected using semi‐structured interviews and were analysed using the approach proposed by Corbin and Strauss.

**Results:**

The findings indicated that nurses' primary concern was for the patient and their own personal/professional identity. Five strategies were found including evaluating situation, identifying error, analysing error and situation, determining the agent for error correction, and reducing error effects. Contextual factors were also highlighted as being important in the error recovery. “Attempting to protect self and patient” was the core category of the study. Nurses' concern about protecting patient life and their own personal/professional identity make them use unprofessional approaches for ER.

## 1 INTRODUCTION

Medical errors (MEs) threaten patient safety and are among the major healthcare challenges throughout the world. They may happen at any stage of care delivery every year, an inadmissible number of patients suffer injuries or die due to unsafe and poor quality healthcare. Most of these injuries are avoidable (Slawomirski et al., 2018). The burden of unsafe care broadly highlights the magnitude and scale of the problem (WHO, 2017). Based on their causes, characteristics and consequences, MEs can be grouped as personal or organizational errors, active or latent errors, (Reason, 1995) and near misses or adverse events (La Pietra et al., 2005).

A study reported that 12% of hospitalized patients experience preventable MEs, 9%–15% of which are severe or lead to death (Panagioti et al., 2019; Robertson & Long, 2018). Studies in Iran also indicated the high prevalence of MEs and reported MEs as a major healthcare challenge (Khammarnia & Setoodehzadeh, 2017). A meta‐analysis revealed that the overall prevalence of MEs in Iran is 50%. The results of this systematic study revealed that the occurrence of medical errors during the care process is high and can be done by any members of the healthcare team and at any time in the process of care delivery in any healthcare environments (Vaziri et al., 2019).

Medical errors are specifically common in intensive care unit (ICU). ICU is a well‐equipped critical care unit with experienced staff for care delivery to patients with critical conditions who need greater support for survival than other patients. Patients in ICU are usually old, have longer hospital stay, receive multiple medications and need sophisticated equipment and drug calculations, and hence, are more at risk for experiencing MEs (Varghese et al., 2018). The results of a study indicated that 46/3% of patients experienced at least one medication error, most of which were clinically important (Ewig et al., 2017) Also, the results of another study revealed that intensive care unit is associated with significant medical errors and adverse events, each of which can lead to an increase in the length of hospitalization of patients in intensive care unit (Ahmed et al., 2015).

Medical errors are associated with many different consequences for patients, healthcare providers and healthcare system. A study indicated that 15% of the total annual healthcare costs pertain to MEs (Slawomirski et al., 2018). As patients in ICU have critical conditions, MEs in ICU may lead to serious consequences such as severe disability and even death (Eulmesekian et al., 2020). Moreover, MEs cause physical and mental problems for healthcare providers (Robertson & Long, 2018; Winning et al., 2018). Therefore, error prevention, management and recovery in ICU are of great importance.

As healthcare providers in the frontline of healthcare delivery, nurses have significant roles in identifying, preventing and correcting MEs (Gaffney, Hatcher, & Milligan, 2016). A study revealed that on average, nurses identified, prevented and corrected one potentially damaging error per week (Vázquez‐Sánchez et al., 2020). The process of error identification, prevention and correction is called error recovery (ER) which happens between error occurrence and damage to patient and aims at reducing or preventing potential and actual damages to patients. ER can be a planned or unplanned process. Planned ER refers to defensive measures to prevent patient damage, while unplanned ER refers to problem‐solving and clinical judgement skills. ER has three consecutive steps, namely error identification, error interception and error correction. Error identification is awareness of error events through techniques such as careful patient identification, healthcare team identification, familiarization with care plan, knowing protocols and policies, use of checklists and use of systematic processes. Error interception refers to nurses' attempt to collect data about error causes, intercept errors or prevent their reoccurrence in future. Strategies for error interception include asking help from others or clarification of orders. Error correction or countering focuses on corrective measures to neutralize error effects such as revising care plan, using a new care plan or referring to specialists. Successful ER prevents patient damage, while unsuccessful ER is associated with adverse consequences (Gaffney, Hatcher, Milligan, & Trickey, 2016; Van der Schaaf et al., 1992). ER is a psychosocial process affected by many different personal factors such as knowledge, judgement, experience and critical thinking skills and social factors such as organizational culture (Theresa Adcock Gaffney, 2015). This is an effective process to ensure patient safety and manage errors and can lead to the development of effective strategies for identifying and correcting errors and empowering organizations for reducing the adverse effects of errors such as disability and death. The ER process emphasizes the importance of professional decision making and clinical reasoning by nurses (Henneman & Gawlinski, 2004).

## 2 BACKGROUND

One of the models for ensuring patient safety is the model developed by Henneman and Gawlinski based on the Eindhoven Model. The Eindhoven Model was originally developed for identifying the root causes of safety‐related incidents in chemical factories in Netherland. Henneman and Gawlinski explained their model using hypothetical scenarios of nursing actions to recover medical errors. The error recovery processes is a sequential three steps, namely error identification, error interruption and error correction (Henneman & Gawlinski, 2004). Despite the importance of ER in preventing MEs and enhancing patient safety, most previous studies dealt mainly with the prevalence and the contributing factors of MEs and less frequently addressed ER. (Bergl et al., 2018; Cheraghi et al., 2012; Farzi et al., 2017; Ghezeljeh et al., 2022; Panagioti et al., 2019; Roth et al., 2015). Moreover, they provided useful information about error characteristics and prevention and their contributing factors but did not give information about nurses' roles and actions in ER. Some of the former studies were also conducted in settings other than ICU (Gaffney, Hatcher, & Milligan, 2016; Gaffney, Hatcher, Milligan, & Trickey, 2016; Henneman et al., 2006) and hence, their findings may not be generalizable to ICU settings. In addition, cultural differences reduce the generalizability of the findings of studies conducted in one sociocultural context to other contexts because culture has key roles in the formation of emotions, experiences and behaviours (Bagheria et al., 2013). Therefore, the present study was conducted to produce more evidence in this area. The aim of the study was to explore the process of ER by ICU nurses.

## 3 METHODS

### 3.1 Design

This qualitative study was conducted in 2018–2020 using the grounded theory approach.

### 3.2 Setting and participants

The study setting was the medical, surgical and neurosurgical ICUs of three teaching hospitals in Tehran, Iran. The research was conducted by three female researchers who had experience working in intensive care unit. Participants were 20 nurses recruited through purposive and theoretical sampling. Eligibility criteria were bachelor's degree or higher in nursing, clinical work experience of at least 2 years and agreement for participation. Initially, a short introductory session was held with each participant to inform the participant about the aim and the methods of the study, invite them to the study, obtain their written consent for participation and determine the time and the place of data collection based on their preferences. The first participant was introduced to the third author by one of her colleagues. Other participants were identified based on the findings in order to enrich the data; with the advancement of class analysis and formation, the process of selecting participants based on theoretical sampling continued and the aim was to collect data based on concepts or themes extracted from previous data. The sampling process continued until the complete saturation of data and classes. After 17 interviews, no new data were obtained, but in order to ensure data saturation, four interviews were conducted with three participants (one was supplementary).

### 3.3 Data collection

Data were collected through semi‐structured interviews held using an interview guide; before each interview, the researcher asked questions about the demographic characteristics of the participants and communicated properly and gained their trust (introducing the researcher as a nurse working in the intensive care unit with 18 years of clinical work experience), paving the way for better and easier interviews. In order to lead the interview forward, the interviews were face‐to‐face, only one interview was conducted in a distance due to the epidemic of the coronavirus and the current situation. After explaining the objectives, the method of studying and obtaining the participant's consent (sending the consent and completing it by the participant) with the participant's information, the interview was conducted online in a software platform (WhatsAPP). During the interview, the talks were recorded using a voice recorder software and then the interview was typed, coded and then analysed as in previous interviews. The guide included broad open‐ended questions such as, “Can you explain about your experiences of errors?” and “What did you or your colleagues do when you noticed an error?” Then, probing questions such as “Can you explain more?” and “Can you clarify this with an example?” were used to collect more detailed data about participants' experiences. All interviews were finished with this question, “Is there anything else about ER you want to add?” Interviews were held by the third author in nurses' break room, head nurse's room or hospital nursing office in the study setting. Sampling and data collection were continued up to data saturation which was achieved after the seventeenth interview. Nonetheless, four additional interviews were held with three participants in order to ensure data saturation. Consequently, 23 interviews were held with 20 participants. The length of the interviews was 30–90 min. All interviews were audio‐recorded using a digital recorder. During data collection, the interviewer (i.e., the third author) wrote her new ideas about the study data and findings as memos and used them for identifying the next interviewee, revising the interview guide, enriching the data and developing the emerging theory. Each interview was immediately transcribed and analysed.

### 3.4 Analysis

The data were analysed concurrently with data collection using the approach proposed by Corbin and Strauss (2015). The five steps of this approach are analysing data for concepts, developing concepts in terms of their properties and dimensions, analysing data for context, bringing process into analysis and integrating categories (Corbin & Strauss, 2015). Analysing data for concepts was started through open coding, in which each interview was transcribed and its transcript was perused several times to obtain a general understanding about its main ideas. Then, the transcript was assessed line by line and word by word. Keywords or sentences were identified and coded, and a list of the primary codes was created. In the second step, constant comparison was performed to develop concepts in terms of their properties and dimensions. Accordingly, the primary codes were compared with each other respecting their similarities and then, codes with conceptual similarity were grouped into primary categories. This process was continued up to the full development of each category in terms of its properties and dimensions. Meanwhile, the third step was taken to analyse the data for context. In this step, the written memos of each interview were reviewed, then a memo summary was made, and subsequently, read several times in order to identify structural and contextual factors affecting the process of ER. For bringing process into analysis in the fourth step, the memos and the raw data were reassessed in order to determine how participants managed their main problems in their context. In the final step, diagrams were drawn, memos were reviewed and a storyline was written in order to identify the core category of the study and develop an integrated theoretical structure. The MAXQDA 10 software was used to organize and manage the data. Consolidated criteria for Reporting Qualitative research was as used to check the quality of our writing (Appendix 1; Tong et al., 2007).

### 3.5 Trustworthiness

Trustworthiness was ensured using the four criteria recommended by Lincoln and Guba, namely credibility, confirmability, dependability and transferability (Lincoln & Guba, 1986). Credibility was maintained through prolonged engagement with the data and participants, member checking and peer checking. In member checking, participants were asked to comment on the congruence between the generated primary codes and their own experiences. In case of any incongruence, the codes were revised according to their comments. In peer checking, two experienced qualitative researchers continuously assessed the accuracy of data analysis in biweekly sessions held together with the study authors. Confirmability and dependability were ensured through documenting all steps of the study in order to provide others with the possibility of tracing research‐related activities. Moreover, transferability was ensured through providing detailed descriptions of participants' experiences.

### 3.6 Ethics

This study has the approval of the Ethics Committee of Iran University of Medical Sciences, Tehran, Iran (code: IR.IUMS.REC.1397.792). All participants were informed about the study aim and methods, were ensured of confidential data management and were free to withdraw from the study at will. Written informed consent was obtained from all participants. The audio files of the interviews were also loaded on a USB drive and were confidentially kept in a safe place.

## 4 RESULTS

Participants were seven male and 13 female nursing staff. The means of their age and ICU work experience were 34.5 (in the 27–52 range) and 6.7 years respectively. Two participants were nursing supervisor, two were head nurse, one was in‐charge nurse and 15 were staff nurse with bachelor's degree.

In total, 11 main categories were generated during data analysis. These categories were on participants' main concern (*n* = 1), strategies for dealing with the main concern (*n* = 5), outcomes of the strategies (*n* = 2) and context (*n* = 3). Participants' main concern was protecting patient life and their own personal and professional identity. They used five main strategies to manage this main concern, namely evaluating situation, identifying error, analysing error and situation, determining the agent for error correction and reducing error effects. Contextual factors which affected their main concern and strategies for main concern management were their professional competencies, organizational safety culture and dominant social culture. The outcomes of participants' strategies were outcomes for patients and outcomes for nurses. These categories are explained in detail in what follows.

### 4.1 Main concern

**FIGURE 1:**
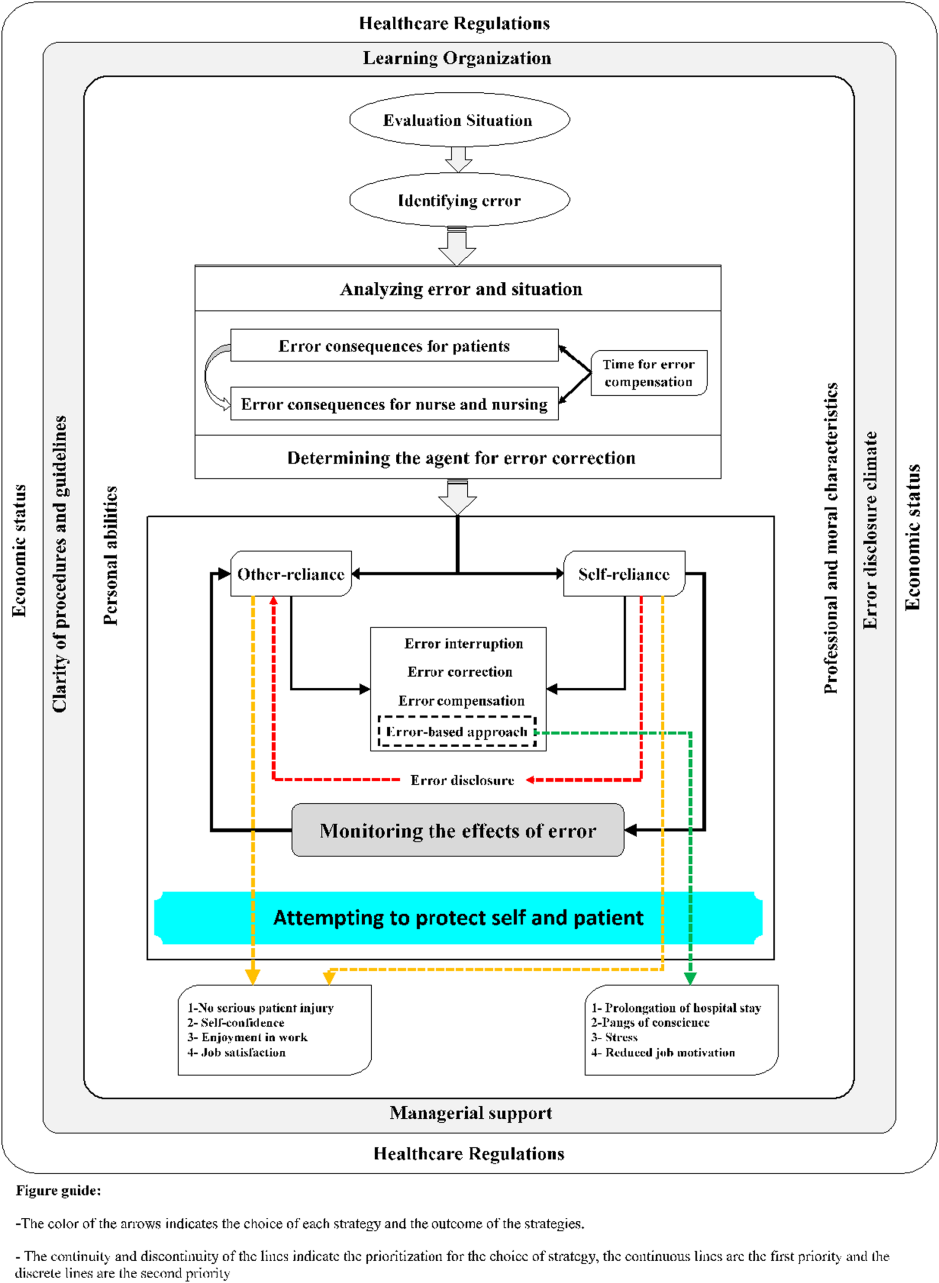
The process of error recovery (ER) in ICU.

The main concern of study participants was protecting patient life and their own personal and professional identity. They were primarily concerned with protecting patient life and hence, made serious efforts to recover errors and took strategies to eliminate, correct and compensate errors. On the other hand, they were concerned with protecting their own personal and professional identity through personal and confidential strategies which could result in the occurrence of new errors. Personal identity refers to individuals' understanding and perceptions of themselves, with which they differentiate themselves from others. Professional identity is a set of sociocultural, mental and philosophical characteristics which are indicative of the unity of the members of a given profession and differentiate that profession from other professional groups. Accordingly, protecting personal and professional identity means defending personal and professional autonomy and individuality against the judgement of others (Figure 1). Factors such as limited managerial support, inappropriate error reporting culture, unsupportive regulations, learning organization and professional and moral characteristics affected participants' choice of strategies.

### 4.2 Strategies for managing the main concern

Participants had taken five main strategies or steps to manage their main concern. These strategies were evaluating situation, identifying error, analysing error and situation, determining the agent for error correction and reducing error effects.

#### 4.2.1 Evaluating situation

The first step of the ER process by nurses was evaluating situation. According to their job specifications and before each intervention, participants evaluated patients and environment through observation, assessment and control. Evaluating situation included strategies such as continuous monitoring of vital signs, assessing the results of laboratory tests, performing hand‐off rounds, requesting anaesthesiology specialists to supervise patients' affairs, intangibly supervising nursing staff by in‐charge nurses and assessing patients and equipment.

I looked at the monitor and found that patient's oxygen saturation was declining. Then, I looked at the patient and found him cyanotic. After that, I checked the oxygen therapy option on the ventilator and noticed that it had not been set accurately. Therefore, we immediately provided the patient with respiratory support. (P. 10)

#### 4.2.2 Identifying error

The second step of the ER process was identifying error. Through evaluating situation, nurses noticed situations which entailed the risk of error and hence, they reflected on the situation, analysed and interpreted it, and compared it with their knowledge and past experiences. For example, they reflected on the reasons for prescribing different medications, appropriateness of their administration routes and appropriateness of their administration doses. Based on their reflections, they used strategies such as reassessing patients' medical records and clarifying the situation for colleagues. Some participants also noted that they sometimes identified some errors incidentally and by chance.

When I saw the medical order, I noticed an error and spoke about it with the doctor. The doctor accepted his error and corrected the order. (P. 7)

#### 4.2.3 Analysing error and situation

After planned or incidental identification of error, nurses analysed the different aspects of the error and the situation such as compensation time, patient conditions, error type and severity, and error consequences for patients, nurses and nursing. Then, they decided on error management based on the results of their error and situation analyses. When the error was not serious or there was no time for error compensation, participants monitored error consequences and made new decisions based on the consequences.

We attempt to correct the error. But, when we can't do anything to correct the error or when the error is not compensable, we monitor the patient for the potential consequences of the error. (P. 3)

#### 4.2.4 Determining the agent for error correction

After identifying the error and analysing error, situation and error consequences for patients and nurses, participants determined the agent for error correction. In other words, they decided to recover the error either individually or with the help of others. They usually attempted to individually recover the error. However, if they found that they could not individually recover the error, they attempted to recover it and reduce its effects in collaboration with others. Most of them selected those colleagues who were confident, were not likely to formally report the error, were collaborative in ER and had adequate knowledge and skills for ER.

When I commit an error, I never talk about it to anyone unless I feel that without requesting help, a serious event or patient death may happen. Then, I tell about the error only to a colleague who is confident and does not report it. (P. 13)

#### 4.2.5 Reducing error effects

Finally, participants employed strategies to eliminate or reduce the effects of error. The three approaches to reduce error effects were error interruption, error correction and error compensation. Depending on error type, these approaches were used either separately or simultaneously. Sometimes, participants used strategies such as verbal warning to interrupt their colleagues' errors. Their strategies for error correction also depended on error type and included accurate documentation of medical orders in the Kardex or changing an intravenous solution. Moreover, they attempted to reduce or compensate error effects through requesting medical consultation or repeating laboratory tests. Sometimes, some participants attempted to individually and confidentially correct or compensate their errors based on their error and situation analysis and using their own past error‐related experiences. However, they might commit new errors in this process of error correction or compensation. Of course, they continuously monitored the effects of their errors and if there was a potential threat to patient life, they might finally decide on error reporting to protect patient life. Although most participants reported error disclosure as a correct strategy, only some of them used it to reduce error effects. They performed error disclosure either formally through documenting the error and filling error‐related forms or informally through telling colleagues or physicians about the error.

Of course, it depends on the error type. For example, when it is a minor error, disclosure is unnecessary. However, when a vancomycin solution which had to be infused over a given period of time was instantly infused and the patient showed flushing reaction and generalized itching, I immediately discontinued the infusion and informed the in‐charge nurse and the doctor. After that, we corrected the error using medications. (P. 13)

### 4.3 Outcomes of the strategies

Participants' strategies for managing their main concern, that is, protecting patient life and their own personal and professional identity, were associated with different outcomes which were categorized as outcomes for nurses and outcomes for patients.

#### 4.3.1 Outcomes for nurses

Depending on error type and ER strategies, the ER process had different positive or negative outcomes for nurses. Examples of the outcomes of strategies such as error disclosure, error elimination and error compensation were personal satisfaction, self‐confidence, enjoyment in work and job motivation. On the other hand, participants' error‐based strategies were associated with negative outcomes such as preoccupation, disinterest in nursing, stress, pangs of conscience and reduced job motivation.

After the error, I independently injected lasix (furosemide) without informing anyone. However, I was preoccupied with the problem for a long time even when I was at home. I was concerned with the possibility of hypotension or any other adverse event for the patient. (P. 15)

#### 4.3.2 Outcomes for patients

Participants' strategies for ER had different outcomes for patients which ranged from no serious patient injury to the prolongation of patient recovery. In overall, timely error identification, error interruption, error disclosure and error compensation were associated with appropriate ER and minimization of injuries to patients. On the other hand, error‐based strategies and confidential error correction were associated with prolongation of hospital stay, need for long‐term antibiotic therapy or prolongation of the extubation process. Nonetheless, most participants noted that they used error‐based strategies due to different reasons such as poor nurse‐physician communications, inappropriate organizational culture and perceived negative outcomes of errors for nurses and nursing.

Of course, my colleague immediately noticed the problem and said that the tube was removed. We immediately provided respiratory support and the doctor rapidly placed a new tube. But, if we hadn't noticed the problem and the patient needed resuscitation, he might have experienced death. Fortunately, no serious problem happened and the patient survived. (P. 11)

### 4.4 Context

Context consisted of factors which accelerated or disrupted the ER process and affected participants' responses to errors. These factors were grouped into three main categories, namely nurses' professional competencies, organizational safety culture and dominant social culture.

#### 4.4.1 Nurses' professional competencies

Nurses' professional competencies, including personal abilities and professional and moral characteristics, facilitated ER. According to the participants, personal abilities such as adequate professional knowledge, great clinical work experience and the ability to predict errors helped them identify, interrupt and correct errors. Examples of professional and moral characteristics which could positively affect the ER process were honesty, conscience, accountability and intra‐ and inter‐professional interactions. Participants noted that patient life in ICU largely depends on nurses' professional performance and hence, nurses' honesty, conscience, accountability, and intra‐ and inter‐professional interactions can facilitate timely error reporting and correction and prevent injuries to patients.

Appropriate practice in ICU largely depends on conscience. You can't report your error unless your conscience commands you to do it. Otherwise, you may simply neglect it and don't take any action for its correction and thereby, nobody may notice your error. Thus, I believe that conscience, responsibility, and ethics are important requirements of work in ICU. (P. 4)

#### 4.4.2 Organizational safety culture

Organizational safety culture can also affect the process of ER. It includes organizational attitudes and perceptions which encourage the correction of safety‐related problems and are indicative of organizational commitment to learn from errors, take actions for improving patient safety, identify potential risks, use the error reporting system, analyse adverse safety‐related events and not to focus on individuals at the time of errors. The organizational safety culture includes aspects such as managerial support, learning from errors, clarity of procedures and guidelines, and error disclosure climate.

We hold safety sessions every three months to analyze errors. Moreover, we hold educational safety‐related sessions for our personnel in order to improve their knowledge and help them share their experiences without any fear. They should be ensured that the system doesn't punish them for their errors and safety‐related interventions aim at improving service quality and patient safety. (P. 18)

#### 4.4.3 Dominant social culture

The dominant social culture can also affect the ER process. This culture includes healthcare regulations and economic status. Economic sanctions against Iran, lack of equipment and resources in healthcare organizations, dominance of medical paternalism in healthcare system, physicians' poor attitude towards nurses, their limited accountability to errors, limited attention to their errors and limited feedback for errors to physicians affect the ER process in Iran.

I think there is no need for correcting the small errors which happen for those patients who don't benefit from critical care in ICU except for life prolongation. For example, I don't change a solution set which becomes unsterile. We are under sanctions and have equipment shortage. (P. 14)

### 4.5 Core category: “attempting to protect self and patient”

The main error‐related concern of nurses in ICU is protecting patient life and their own personal and professional identity. In order to manage this concern, nurses in ICU carefully evaluate their situation, assess patients, supervise others and reflect on situation to identify potential or actual errors. After error identification, they analyse the identified error and situation, the time for error compensation, and the outcomes of error for patients and nurses. Their error‐related decisions largely depend on potential and actual consequences of error and their actions. Despite the availability of different options for error disclosure and correction, nurses may decide not to report their errors, resort to error‐based approaches, and attempt to individually correct errors based on their personal knowledge and experiences or through asking their colleagues' help. The results of their error analysis respecting the effects of errors on patient life and their own personal and professional identity largely affect their error‐related decisions. In other words, they attempt to recover their errors through strategies that protect patient life and their own personal and professional identity. Accordingly, the ER process can be called as the process of “Attempting to protect self and patient.” When nurses use appropriate ER strategies, the outcomes of the ER process are job satisfaction, job motivation, and self‐confidence. However, when they use error‐based approaches for ER, the outcomes of the ER process are feelings of fear, anxiety, preoccupation and limited motivation (Figure 1).

## 5 DISCUSSION

This study explored the process of ER by ICU nurses. Findings revealed that this process is the process of “Attempting to protect self and patient.” This process includes ER strategies, ER outcomes and context, which are discussed in what follows.

### 5.1 ER strategies

The first ER strategy of the study participants was evaluating situation, including patient, changes in patient's conditions, patient's environment and activities, in order to identify potential or actual errors. A former study also indicated that error identification in neonatal ICU was performed using equipment alarms, nurses' direct observation and patients' medical record data (Babaeipouya et al., 2017). Therefore, data collection and situational analysis through observation and patient monitoring are considered as the most important strategy for error identification. Similarly, a conceptual model holds that the beginning of ER is spontaneous or systematic error perception, that is, understanding the situation or the problem. Error perception relies on data collection from different sources. Knowledge and expertise are helpful in this step, though knowledgeable and skilful individuals may sometimes be unable to accurately perceive error (Patel et al., 2011). Audiovisual tools such as monitoring devices and checklists can be used to assess the signs and evidence of errors, reduce error consequences and improve patient safety Managers need to repeatedly highlight the importance of continuous patient monitoring in ICU. Moreover, as most errors happen during shift turnover, hospital managers need to provide their staff with standard handover techniques, protocols and tools such as the Situation, Background, Assessment, Recommendation (SBAR) tool in order to strengthen professional relationships, reduce errors and improve patient safety (Stewart, 2016).

The second step of the ER process in the present study was error identification. In this step, nurses used different strategies for error identification, including re‐evaluating patients' medical records, comparing Kardex data with medical orders, and providing explanations or clarifications to colleagues. A study into ER strategies revealed that nurses used strategies such as assessing patients, recognizing people with significant roles in ER, knowing the plan of care, surveillance, knowing policies and procedures, double‐checking, questioning and employing systematic processes (Henneman et al., 2010). In the present study, knowing the plan of care strategy was not identified as an ER strategy. This contradiction is attributable to the difference between these two studies in terms of their population and sociocultural context. Understanding and being aware of errors can be planned or spontaneous. In the present study, what made the nurses' alert to the upcoming situation and the use of mentioned strategies was the previous knowledge and trainings, and the past experiences. In some cases, errors were identified either by accident or based on nurses' intuition, which were interpreted as the patient's luck. Therefore, error recovery, prevention of adverse outcomes and improvement of patient safety will occur due to early identification of error evidence. Timely identification of errors is as important as error reduction strategies (Patel et al., 2011).

Analysing error and situation was the third step of the ER process in the present study. Participants reported that before taking any action, they analysed the outcomes of errors and error‐related actions for patients and nurses and then, made error‐related decisions based on the results of their analysis. Nurses in ICU have certain criteria for decision making about errors. They may not consider many consequence‐free events as errors and hence, may underestimate the errors that may have serious consequences. Errors in ICU cause the most serious consequences and risks for patients who are critically ill and are vulnerable to the adverse effects of errors (Roque et al., 2016). A study indicated that error analysis is a type of decision making which nurses perform following the occurrence of errors and noted that they decide on error disclosure only when the intended patient is conscious, their family members are present, or there is a probability of injury to patient and legal consequences for nurses (Crigger & Godfrey, 2014). In line with our findings, another study reported that one of the steps of the error management process was cognitive step, in which nurses assessed the occurred error and then, managed or reported it (Valee et al., 2014). Weighing is a permanent process that was performed before and after the selection of corrective actions. The results of a study by Crigger and Meek (2007) was in line with the present study and revealed that weighing is the kind of decision that some nurses make after an error occurs. During the weighing phase, participants determined whether the error was real or false. The primary indicators of a real error is whether that error has resulted in harm to the patient? The reasons nurses cited for exposing errors included the possibility of harm to the patient and the existence of legal consequences (Crigger & Meek, 2007). One of the important points in the process of error recovery was understanding and signifying the error occurred and also its consequences by nurses. Therefore, it is important that the nursing managers pay more attention to nurses' experiences of errors or their peers' in the field by holding refresher courses about the concept of error and its consequences, in order to change the way it is perceived. On the other hand, the consequences affecting nursing were other important issues as well, in general nurses are afraid of tarnishing their professional reputation and avoid being recognized as wrong persons in the organization (Ghezeljeh et al., 2021). Error outcome is one of the key elements determining error recovery behaviours between medical staff especially nurses. Managers should try to create an atmosphere of trust in the field of errors and report them in these wards in order to show appropriate response to people who have reported their errors so that nurses show more willingness to report theirs, therefore, leading to effective error recovery.

The final steps of the ER process in the present study were determining the agent for error correction and reducing error effects. Evaluating and identifying error‐related evidence are not adequate for appropriate ER; rather, error identification should be followed by reducing error effects. Our participants individually or collectively employed interventions to interrupt or correct their errors and reduce their effects. Similarly, a study showed that nurses avoided disclosing their errors and attempted to manage them either independently or with their colleagues' help. Then, they assessed patient's conditions in order to ensure patient safety and life. They disclosed their errors only when their personal or collective ER measures were ineffective (Nasrabadi et al., 2017). However, some personal or collective ER measures may be non‐standard and cause more damage to patients. Researchers stated that sometimes taking actions alone was not in accordance with the standards and, therefore, they chose their concept of self‐ action, but in the present study, measures that were not in accordance with the standards could be taken individually or in groups and these measures were named error‐based measures. Another study into the clinical instructors' experiences of managing students' errors stated that instructors independently took measures to correct their students' errors, monitored afflicted patients' conditions for potential adverse effects of errors and informed colleagues and nurses if the adverse effects of errors appeared. That study also specified that when errors had life‐threatening effects on patients, instructors and nurses informed physicians to take life support measures and prevent life‐threatening conditions (Shahoei et al., 2019). Error correction is among the professional error‐related responsibilities of nurses; however, inappropriate error correction measures contradict the ethical principle of non‐maleficence and may cause injuries to patients (Olson & Stokes, 2016). The results of a study revealed that the five steps of decision making for reporting errors were error identification, living the error and taking immediate or delayed measures for its correction, formal or informal error reporting, living the aftermath of the error, and feeling conscientious. Nurses in that study used strategies such as apology and clarification for their disclosed errors (Koehn et al., 2016), while our participants reported no use of such strategies or used strategies which were not in their job specifications. We called this approach to ER as error‐based approach because they used unauthorized strategies which could result in adverse consequences for patients or nurses. Although these measures could have been taken in groups, they were individual‐oriented actions. It should be considered that healthcare and hospital managers should give clear guidelines to nurses so that when they encounter errors, do not face ethical challenges and expose errors without any obstacles, and to provide nurses with the necessary trainings in the correct and professional direction to errors recovery, both individuals and groups, in order to make error recovery successful. Error reduction occurs when nurses understand the causes of errors, identify them correctly and rely on evidence‐based interventions (Motazedi et al., 2019).This difference between these two studies may be due to our participants' fear over stigma and loss of reputation Stigma has different influences in societies with different cultures. Therefore, in order to take professional actions in accordance with the ethical principles mentioned by the American Nursing Association, in addition to emphasizing on maintaining and promoting patient safety, it is necessary to consider the culture of apology and explaining the process of error to the patient or patient's companions in nursing educational and clinical courses by planners and policymakers in this field.

### 5.2 Context

Findings revealed that contextual factors which affected the process of ER included professional competencies, organizational safety culture and dominant social culture. Participants' experiences indicated that nurses' knowledge and clinical skills affected error identification, elimination and correction. Participants used their competencies to recover not only their own errors but also their colleagues' and physicians'. In line with these findings, an earlier study reported that nurses with more clinical experience had greater knowledge and information about guidelines, were more able to identify, interrupt and correct actual and potential errors, and hence, had better ER performance (Wilkinson et al., 2014). The results of the Gaffney, Hatcher, & Milligan (2016), Gaffney, Hatcher, Milligan, & Trickey (2016) study were in line with our study and indicated that error recovery is directly related to the level of clinical education and skills, higher level of education and expertise, clinical judgement, critical thinking and problem solving facilitates, previous experiences in a situation of identifying and retrieving medical errors. Therefore, nursing managers should consider that well‐educated nurses with clinical experience should be used for intensive care unit and also in addition to error prevention, provide the ground for effective recovery of errors by holding training courses in different fields of care and treatment. We also found inter‐professional interactions as a contextual factor affecting the ER process. This is in line with the findings of a former study which reported that trust and respect among healthcare providers enhance their satisfaction, improve their sense of responsibility towards patients, promote their involvement in care delivery, and thereby, improve patient safety and care quality (Häggström et al., 2017). Inter‐professional collaboration and interactions not only improve staff's satisfaction and reduce their stress but also promote their adherence to patient safety standards (Vaismoradi et al., 2020). Care delivery in ICU is a multidimensional process that needs interprofessional understanding, collaboration and interaction Healthcare providers' participatory practice and effective interpersonal interactions improve their understanding of situations, facilitate error identification and management, and improve care quality and patient survival (Rosen et al., 2018). Contrarily, poor interprofessional collaboration, particularly between nurses and physicians, can lead to error ignorance. Therefore, fostering a collaborative and interdisciplinary care delivery culture and improving healthcare providers’ communication skills through educational workshops are necessary to improve their ER skills.

Study findings also revealed that another factor affecting the ER process was the dominant social culture which consisted of economic factors, formal structure of authorization and the dominance of medical paternalism in healthcare. Similarly, a study reported that the economic status of healthcare organizations can affect patient care delivery and patient safety (Akinleye et al., 2019). However, another study proved that while personal and organizational factors affect ER, social culture and economy were not among factors affecting ER (Gaffney, Hatcher, & Milligan, 2016; Gaffney, Hatcher, Milligan, & Trickey, 2016). This difference between the findings of the studies is attributable to the differences in their sociocultural contexts.

Study findings also indicated organizational safety culture as another contextual factor affecting the ER process. A positive organizational safety culture is associated with greater likelihood of error disclosure and also healthcare providers' reports can improve patient safety (Alijanzadeh et al., 2015). Such culture helps healthcare providers prioritize patient safety and includes components such as organizational learning, teamwork, open communications and punishment‐free feedback. A study showed that encounter with errors was a traumatic process and hence, individuals evaluated different factors before deciding whether to disclose or hide their errors. That study showed that adequate perceived support and confidence in punishment‐free feedback helped individuals effectively communicate and manage their errors and promoted their learning from errors. It was important to note that remembering the error situation long after the incident could be helpful in learning and identifying similar situations (Zieber & Williams, 2015).

Non‐punitive error disclosure and learning from errors are among the goals of patient safety in healthcare organizations in Iran (MOHME, 2017). Nonetheless, some managers still blame and punish staff for errors, hold misconceptions about errors, focus on individuals rather than errors and give no constructive error‐related feedback to staff. These beliefs and behaviours result in poor error disclosure in some healthcare settings and by some healthcare providers (Mollaei et al., 2018), confidential error management, occurrence of new errors during error management, and prolongation of patient ICU stay. Healthcare policymakers need to employ systemic approaches to error management, avoid blame and punishment for errors, promote learning from errors and create a positive organizational safety culture in healthcare organizations in order to facilitate ER.

### 5.3 Outcomes of the strategies

Our findings showed that ER or error non‐disclosure had different positive or negative outcomes for patients and nurses. Error disclosure and ER were associated with satisfaction, motivation, self‐confidence and positive error‐related experiences, while error non‐disclosure was associated with fear, anxiety, preoccupation and reduced motivation for nurses. Healthcare providers usually experience a wide range of negative emotions such as guilt, doubt, embarrassment, despair, self‐blame, fear and insufficiency when they face errors. A study showed that nurses in ICU experienced senses of upset, guilt and blame immediately after errors and experienced different positive and negative feelings such as satisfaction after error management (Valee et al., 2014). Another study showed that ineffective error correction caused nurses pain, concern and preoccupation for long periods of time (Crigger & Meek, 2007). The Hurley et al. (2008) study also revealed that recovery can be caused by consequences such as no damage to the patient (Near miss) or adverse events. Subsequently, either of these two outcomes can cause positive or negative feelings such as hopelessness in nurses or positive feelings if the error was resolved. It seems that nursing managers can lead their positive feelings in the correct direction of error recovery by engaging more and giving responsibilities to nurses at the time of the occurrence of errors, otherwise this encourages the nurses to resolve errors in the future. Post‐error damages to healthcare providers turn them into the secondary victims of errors and give them feelings such as failure and insufficiency. Therefore, healthcare policymakers and hospital managers need to develop effective strategies for effective error prevention and ER in order to reduce the negative effects of errors.

### 5.4 Implications

According to the purpose and type of the study, the present results better displayed the process of error recovery by nurses in intensive care units and the related factors of situations, barriers and facilitators in its context were better identified. Therefore, its barriers and facilitators can be a guide for nursing managers and policymakers which can help give appropriate solutions to create a correct and complete process for error recovery by nurses. Considering that one of the important and influential steps on the decision‐making of intensive care unit nurses is comprehensive analysis and evaluation of different situations and their consequences for patients and nurses in relation to nurses' perception of the concept of nursing errors. Nurses considered errors as a damaging action and inconsistent with the patient's health and contrary to the academic learnings which should be considered by hospital educational policymakers and by further explaining the job description and standards required, holding workshops with the concept of medical errors and error recovery in order to familiarize intensive care unit nurses with this concept and applying it at work, consequently, this will nurses' attitudes towards the nature of errors should be changed. It is suggested that meetings be held in hospitals and medical centres so that nurses in different wards, including the intensive care unit, share their experiences about error recovery and take steps to prevent, identify and recover errors.

### 5.5 Limitations and recommendations

Considering the fact that this study was conducted in educational hospitals it can be different for intensive care unit nurses in private hospitals due to its context, so other future studies in this regard on nurses working in private hospitals and adaptation of the present findings and those of the future can be effective in understanding this problem and conducting future plans. Moreover, the present study is the first study with a grounded theory methodology about error recovery and factors affecting it, so further studies are needed to understand how to recover the errors.

## 6 CONCLUSION

This study suggests that the process of ER by nurses in ICU is the process of “struggle to protect self and patient” which consists of five main strategies, namely evaluating situation, identifying error, analysing error and situation, determining the agent for error correction and reducing error effects. This process is affected by different contextual factors, namely nurses' professional competencies, organizational safety culture and dominant social culture and results in positive and negative outcomes for nurses and patients. Nursing managers and policymakers can use the findings of this study to develop strategies for facilitating appropriate ER and creating a positive organizational safety culture to improve patient safety and care quality. Hospital authorities are also recommended to implement educational programs on patient safety, ER and principles of ethical practice such as veracity and non‐maleficence.

## AUTHOR CONTRIBUTIONS

Coordination with nurses and conducting interviews was done by the third researcher, but 3 authors participated in all stages of conducting and writing the research.

## FUNDING INFORMATION

This study has the approval of the Ethics Committee of Iran University of Medical Sciences, Tehran, Iran (code: IR.IUMS.REC.1397.792) and is financially supported by Iran University of Medical Sciences.

## CONFLICT OF INTEREST STATEMENT

Authors declared no conflict of interests and all of them had meaningful contribution to the study.

## ETHICS STATEMENT

This material is the authors' own original work, which has not been previously published elsewhere. This article sprang from a PhD dissertation in the Faculty of Nursing and Midwifery of Iran University of Medical Sciences, and was approved by Ethics Committee of Iran University of Medical Sciences (code: IR.IUMS.REC.1397.792). All participants were informed about the study aim and methods, were ensured of confidential data management, and were free to withdraw from the study at will. Written informed consent was obtained from all participants. The audio files of the interviews were also loaded on a USB drive and were confidentially kept in a safe place.

## Data Availability

The data that support the findings of this study are available from the corresponding author [F. K. L], upon reasonable request.

## APPENDIX 1

Consolidated criteria for reporting qualitative research (COREQ): a 32‐item checklist for interviews and focus groups.

**Table:**

Item	Guide questions/description	Page
**Domain 1: Research team and reflexivity personal characteristics**		
1. Interviewer/facilitator	Which author/s conducted the interview or focus group? Response: F.K.L	
2. Credentials	What were the researcher's credentials? E.g. PhD, MD Response: N.GH is, professor, PhD. M.AS.F is, Professor, PhD, and F.K.L, is PhD Candidate of nursing	
3. Occupation	What was their occupation at the time of the study? Response: N.GH and M.AS.F, are working Nursing Care Research Center, School of Nursing and Midwifery Iran University of Medical Sciences, F.K.L, is PhD Candidate of nursing, Nursing Care Research Center, School of Nursing and Midwifery Iran University of Medical Sciences	
4. Gender	Was the researcher male or female Response: Female	4
5. Experience and training	What experience or training did the researcher have? Response: All researchers had experience working in ICU and, are members of Nursing Care Research Center	4
**Relationship with participants**		
6. Relationship established	Was a relationship established prior to study commencement?	4
7. Participant knowledge of the interviewer	What did the participants know about the researcher? For example, personal goals, reasons for doing the research	4
8. Interviewer characteristics	What characteristics were reported about the interviewer/facilitator? For example, Bias, assumptions, reasons and interests in the research topic Response: All the research team members had experience working in ICU.	
**Domain 2: Study design** **Theoretical framework**		
9. Methodological orientation and Theory	What methodological orientation was stated to underpin the study? For example, grounded theory, discourse analysis, ethnography, phenomenology, content analysis	4
Participant selection 10. Sampling	How were participants selected? For example, purposive, convenience, consecutive, snowball	4–5
11. Method of approach	How were participants approached? For example, face‐to‐face, telephone, mail, email Response; face‐to‐face and telephone	5
12. Sample size	How many participants were in the study?	5
13. Non‐participation	How many people refused to participate or dropped out? Reasons?	NO
**Setting**		
14. Setting of data collection	Where was the data collected? For example, home, clinic, workplace	5
15. Presence of non‐participants	Was anyone else present besides the participants and researchers?	No
16. Description of sample	What are the important characteristics of the sample? For example, demographic data, date	6
Data collection 17. Interview guide	Were questions, prompts, guides provided by the authors? Was it pilot tested? Response: yes	5
18. Repeat interviews	Were repeated interviews carried out? If yes, how many? Response: Yes, 3 times	4
19. Audio/visual recording	Did the research use audio or visual recording to collect the data? Response: Audio recording	5
20. Field notes	Were field notes made during and/or after the interview or focus group? Response: Yes	5
21. Duration	What was the duration of the interviews or focus group? Response: yes	5
22. Data saturation	Was data saturation discussed? Response: yes	5
23. Transcripts returned	Were transcripts returned to participants for comment and/or correction? Response: yes	6
**Domain 3: analysis and findings data analysis**		
24. Number of data coders	How many data coders coded the data? Response:3coder	4
25. Description of the coding tree	Did authors provide a description of the coding tree?	Yes
26. Derivation of themes	Were themes identified in advance or derived from the data? Response: Themes were derived from the data	6–12
27. Software	What software, if applicable, was used to manage the data? Response: MAXQDA‐10 was used to manage and organize the data.	6
28. Participant checking	Did participants provide feedback on the findings?	6
Reporting		
29. Quotations presented	Were participant quotations presented to illustrate the themes/findings? Was each quotation identified? For example, participant number	6–11
30. Data and findings consistent	Was there consistency between the data presented and the findings? Response: yes	6–12
31. Clarity of major themes	Response: yes ere major themes clearly presented in the findings?	6–12
32. Clarity of minor themes	Is there a description of diverse cases or discussion of minor themes?	6–12
